# Certolizumab pegol plus methotrexate 5-year results from the rheumatoid arthritis prevention of structural damage (RAPID) 2 randomized controlled trial and long-term extension in rheumatoid arthritis patients

**DOI:** 10.1186/s13075-015-0767-2

**Published:** 2015-09-10

**Authors:** Josef S. Smolen, Ronald van Vollenhoven, Arthur Kavanaugh, Vibeke Strand, Jiri Vencovsky, Michael Schiff, Robert Landewé, Boulos Haraoui, Catherine Arendt, Irina Mountian, David Carter, Désirée van der Heijde

**Affiliations:** Medical University of Vienna and Hietzing Hospital, Vienna, Austria; Karolinska Institute, Stockholm, Sweden; UCSD, San Diego, CA USA; Biopharmaceutical Consultant, Portola Valley, CA USA; Rheumatology Institute, Prague, Czech Republic; University of Colorado, Denver, CO USA; Academic Medical Centre, Amsterdam, Netherlands; Centre Hospitalier de l’Université de Montréal, Montreal, Canada; UCB Pharma, Brussels, Belgium; Leiden University, Leiden, Netherlands; Department of Medicine, Medical University of Vienna and Hietzing Hospital, Vienna, Austria

## Abstract

**Introduction:**

As patients with rheumatoid arthritis (RA) receive treatment with anti-tumour necrosis factors over several years, it is important to evaluate their long-term safety and efficacy. The objective of this study was to examine the safety and benefits of certolizumab pegol (CZP)+methotrexate (MTX) treatment for almost 5 years in patients with RA.

**Methods:**

Patients who completed the 24-week Rheumatoid Arthritis Prevention of Structural Damage (RAPID) 2 randomized controlled trial (RCT; NCT00160602), or who were American College of Rheumatology (ACR) 20 non-responders at Week 16, entered the open-label extension (OLE; NCT00160641). After ≥6 months treatment with CZP 400 mg every two weeks (Q2W), dose was reduced to 200 mg Q2W, the approved maintenance dose. Safety data are presented from all patients who received ≥1 dose CZP (Safety population, n=612). Efficacy data are presented to Week 232 for the intent-to-treat (ITT, n=492) and Week 24 CZP RCT Completer (n=342) populations, and through 192 weeks of dose-reduction for the Dose-reduction population (patients whose CZP dose was reduced to 200 mg, n=369). Radiographic progression (modified total Sharp score change from RCT baseline >0.5) to Week 128 is reported for the Week 24 CZP Completers.

**Results:**

In the RCT, 619 patients were randomized to CZP+MTX (n=492) or placebo+MTX (n=127). Overall, 567 patients (91.6%) entered the OLE: 447 CZP and 120 placebo patients. Of all randomized patients, 358 (57.8%) were ongoing at Week 232. Annual drop-out rates during the first four years ranged from 8.4–15.0%. Event rates per 100 patient-years were 163.0 for adverse events (AEs) and 15.7 for serious AEs. Nineteen patients (3.1%) had fatal AEs (incidence rate=0.8). Clinical improvements in the RCT were maintained to Week 232 in the CZP Completers: mean Disease Activity Score 28 (Erythrocyte Sedimentation Rate) change from baseline was −3.4 and ACR20/50/70 responses 68.4%/47.1%/25.1% (non-responder imputation). Similar improvements observed in the ITT were maintained following dose-reduction. 73.2% of CZP Completers had no radiographic progression at Week 128.

**Conclusions:**

In patients with active RA despite MTX therapy, CZP was well tolerated, with no new safety signals identified. CZP provided sustained improvements in clinical outcomes for almost 5 years.

**Trial registration:**

ClinicalTrials.gov, NCT00160602 and NCT00160641. Registered 8 September 2005.

**Electronic supplementary material:**

The online version of this article (doi:10.1186/s13075-015-0767-2) contains supplementary material, which is available to authorized users.

## Introduction

Anti-tumor necrosis factors (anti-TNFs) are well-established therapies for the treatment of active rheumatoid arthritis (RA) [1]. Nonetheless, continued research is needed to evaluate their long-term efficacy and safety in RA patients. Certolizumab pegol (CZP) is a PEGylated anti-TNF Fab employed for treatment of adults with moderate-to-severe RA. Results from the Rheumatoid arthritis prevention of structural damage (RAPID) 2 randomized controlled trial (RCT) demonstrated rapid and significant improvements in signs and symptoms of RA after 24 weeks of treatment, using a loading dose of 400 mg at weeks 0, 2, and 4 followed by CZP 200 mg or 400 mg every other week (Q2W) + methotrexate (MTX) [2].

Patients who completed the 24-week RCT or who withdrew at week 16 due to lack of response were eligible to enter the open-label extension (OLE). In the OLE, all patients initially received CZP 400 mg Q2W + MTX for ≥6 months, followed by a dose reduction to 200 mg Q2W, the approved maintenance dose of CZP. The primary objective of this paper is to assess the long-term safety and efficacy of CZP + MTX treatment up to 232 weeks (approximately 4.5 years) in patients with active RA. Additionally, we report efficacy analyses for patients who experienced dose reduction during the OLE and explore whether the timing of the initial response to CZP or lack of response upon OLE entry had an impact on long-term outcomes.

## Methods

### Study design

The RAPID 2 OLE (NCT00160641) was conducted at 68 centers across 13 countries; the design of the phase 3 RAPID 2 RCT study has been reported previously [2]. In the RCT, patients were randomized 2:2:1 to receive one of two regimens of subcutaneous (sc) CZP (200 mg or 400 mg Q2W) + MTX following administration of the loading dose (400 mg at weeks 0, 2 and 4), or placebo Q2W + MTX for 24 weeks. In the OLE, all patients initially received CZP 400 mg Q2W + MTX for at least 6 months, including patients originally randomized to 200 mg Q2W in the RCT. Following a protocol amendment, CZP dose was then reduced to 200 mg sc Q2W, the approved maintenance dose of CZP. This amendment was driven by data from RAPID 1 [3] and RAPID 2 [2], which demonstrated that 400 mg Q2W provided no additional benefit compared with 200 mg Q2W.

Both studies were conducted in accordance with International Conference on Harmonisation-Good Clinical Practice requirements and the ethical principles that have their origin in the principles of the Declaration of Helsinki. Informed patient consent and all appropriate ethical committee approvals were obtained for both studies. Full details of the ethical bodies and committees that approved the study for each center are provided in “Acknowledgements”.

### Patients

Inclusion and exclusion criteria for the RCT are reported in the primary publication [2]. In brief, eligible patients had active RA of ≥6 months’ duration prior to screening and had received MTX therapy for ≥6 months (stable dosage of ≥10 mg/week for ≥2 months prior to baseline). Changes in MTX dose were permitted during the OLE if clinically indicated, but MTX discontinuation was not. Disease-modifying anti-rheumatic drugs (DMARDs) other than MTX were not permitted during the RCT, but were allowed during the OLE; other biological therapies were prohibited throughout.

Two groups of patients were eligible to enter the OLE: (1) CZP or placebo patients who completed the 24-week RCT and (2) CZP or placebo patients who were American College of Rheumatologists (ACR)20 non-responders at both weeks 12 and 14 in the RCT, and were required to withdraw from the RCT at week 16.

### Patient retention

Kaplan-Meier analysis was used to estimate patient retention in the CZP intention-to-treat (ITT) population. This included patients who withdrew for any reason compared with those who withdrew due to an adverse event (AE) or lack of efficacy only. The annual dropout rate in the CZP ITT population for each year of the study is presented as a percentage and was calculated as follows: (number of patients on treatment at the beginning of the year who were no longer on treatment at the end of the year)/(total number of patients on treatment at the beginning of the year) × 100.

### Safety assessment and analysis

The primary objective of the OLE was to assess the long-term safety of CZP + MTX treatment in patients with active RA. Combined safety data from the RCT and OLE are presented. The safety population included all patients who received ≥1 dose of CZP in either the RCT or OLE. Data are reported from the time of first CZP exposure until last visit or patient withdrawal, plus 12-weeks follow up (i.e., five times CZP half-life + 14 days injection, or six times CZP half-life). AEs and serious AEs (SAEs) were assessed at every visit and classified by system organ class and preferred term according to the MedDRA dictionary v9.0. AEs are reported as exposure-adjusted event rates (ER) and incidence rates (IR) per 100 patient-years (PYs). The most frequent AEs by preferred term (ER >4.0 per 100 PYs) are also reported.

### Concomitant medication use

Concomitant medication use was measured at each visit throughout the OLE and coded using the World Health Organization Drug Dictionary (WHO-DD) 2004/Q4. Concomitant medications were those starting on or after the date of first study drug administration in the RAPID 2 RCT and on or before the last on-study visit in the RAPID 2 OLE. Use of concomitant medications is presented for all patients who entered the OLE and summarized by patients taking any non-steroidal anti-inflammatory drug (NSAID), any corticosteroid, any selective immunosuppressant, any anti-malarial drug, or any DMARD other than MTX.

### Efficacy evaluations

Secondary objectives of the RAPID 2 OLE were assessment of the continued effectiveness of CZP + MTX treatment in patients with active RA, including analysis of the impact of treatment on physical wellbeing and quality of life. Efficacy data and patient-reported outcomes (PROs) are presented from RAPID 2 RCT baseline to approximately 4.5 years of CZP treatment (220–232 weeks) for the following populations: (1) all patients randomized to CZP (400 mg or 200 mg) in the RCT (CZP ITT population), (2) patients randomized to CZP (400 mg or 200 mg) who completed the 24-week RCT and enrolled in the OLE (week 24 CZP completers) and (3) patients randomized to placebo who completed the 24-week RCT and enrolled in the OLE (week 24 placebo completers).

Disease activity score in 28 joints (erythrocyte sedimentation rate) (DAS28(ESR)) [4], ACR20/ACR50/ACR70 response rates [5], health assessment questionnaire-disability index (HAQ-DI) [6], patient’s global assessment of disease activity (PtGA) (using 100 mm visual analog scale (VAS)), and patient’s assessment of arthritis pain (VAS) were measured at entry to OLE, at weeks 36, 48, 64, and 76, and then every 12 weeks throughout the OLE. Fatigue (VAS) [7] and short-form-36 (SF-36) [8] were assessed at baseline, weeks 36, 48, and 76, and then every 24 weeks throughout the OLE. Continuous measures are reported as absolute values or mean changes from RCT baseline. DAS28(ESR) <2.6 remission is also reported. For PROs, minimally clinically important differences (MCID) were defined as decrease of ≥0.22 points from baseline in HAQ-DI scores [9, 10], improvements ≥2.5 from baseline in SF-36 physical component summary (PCS) scores [11] and improvements of ≥10 mm in VAS scales [12].

Clinical disease activity index (CDAI) remission (CDAI ≤2.8), simplified disease activity index (SDAI) remission (SDAI ≤3.3) and ACR-European League Against Rheumatism (EULAR) 2011 Boolean-based remission criteria [13] using four variables (Boolean 4: swollen joint count (SJC) and tender joint count (TJC) ≤1, C-reactive protein (CRP) ≤1 mg/dL and PtGA ≤1) and three variables (Boolean 3: SJC and TJC ≤1 and PtGA ≤1) were analyzed post hoc in the CZP ITT and week 24 CZP completer populations. The effects of CZP dose reduction in patients from the RAPID 2 CZP ITT population who initially received CZP 400 mg Q2W + MTX were analyzed post hoc by efficacy analyses (ACR20/50/70 and DAS28[ESR]) over 192 weeks exposure following dose-reduction.

Patients treated with CZP who completed the RCT were further stratified into early and late responders. Early responders were defined as clinical responses at week 12; later responders as responses at week 24, but not week 12. Responses were defined in two ways: ACR20 response (as defined in the protocol) or decrease from baseline in DAS28(ESR) ≥1.2. Efficacy measures (ACR50, HAQ-DI, DAS28(ESR)) were compared post hoc in early vs late responders. Similar analyses also compared early and late responders with the ACR20 non-responder population, which included patients treated with CZP who withdrew at week 16 due to failure to achieve an ACR20 response, and week 24 CZP completers who were recorded as not achieving an ACR20 response at OLE entry.

### Assessment of radiographic progression

Radiographs of hands and feet were performed at RCT baseline and endpoint and/or entry to OLE and weeks 48, 100 and 128. Total and mean changes from baseline in modified total Sharp score (mTSS) [14] and percentage of patients with no radiographic progression (mTSS change from RCT baseline ≤0.5) are presented for week 24 CZP and placebo completer populations. Additional post hoc radiographic analyses (mTSS) were compared in early vs late responders (response defined by decrease from baseline in DAS28(ESR) ≥1.2).

### Statistical analyses

Efficacy results were analyzed descriptively with mean and standard deviations for continuous variables and with counts and percentages for binary variables. Observed data are presented alongside imputed data for all efficacy variables in the CZP completer, CZP ITT and dose-reduction populations. Non-responder imputation (NRI) was used to assess ACR responses. Additional analyses assessed ACR response using mixed model repeated measures (MMRM) imputation; the individual components were imputed using MMRM prior to calculation of response. Modified NRI was used to assess DAS28(ESR) low disease activity (LDA) and DAS28(ESR) remission for timing of response analyses. Missing mTSS data were imputed by linear extrapolation if at least baseline and one follow-up radiograph were available.

LOCF/NRI was used to assess DAS28(ESR), CDAI, SDAI and ACR-EULAR Boolean-based remission in the CZP completer and ITT populations. For LOCF/NRI analyses, missing values during the RCT and missing values for patients not entering the OLE were imputed by carrying forward the last available post-baseline composite score. For patients entering the OLE, missing values during the OLE due to patient withdrawal (AE or lack of efficacy) or data exclusion after rescue medication use were imputed as non-remitters, whereas missing values due to other withdrawal reasons, study completion, or a missing assessment were imputed by carrying forward the last available post-RCT entry score. If still missing, then the value was imputed as non-remitter.

MMRM imputation was used for all other efficacy variables; the MMRM model specifications had visit, gender, region, rheumatoid factor, and treatment in the original RCT as fixed effects. If the variable was changed from baseline, the baseline values were added as covariates. The visits of each patient were the repeated measure in the model. Additional analyses for all continuous efficacy variables were assessed using LOCF imputation. For PRO MCIDs, LOCF/NRI analysis was used.

Binary and continuous variables are presented using NRI and MMRM imputed values, respectively, because NRI is a more conservative imputation method than MMRM for imputing the components of responder variables. All analyses not shown here are presented as part of the “Supplementary materials” (Additional file 1). Where the table or figure number is not indicated, data shown are present in the text only.

## Results

### Patient disposition and baseline demographics

**Fig. 1:**
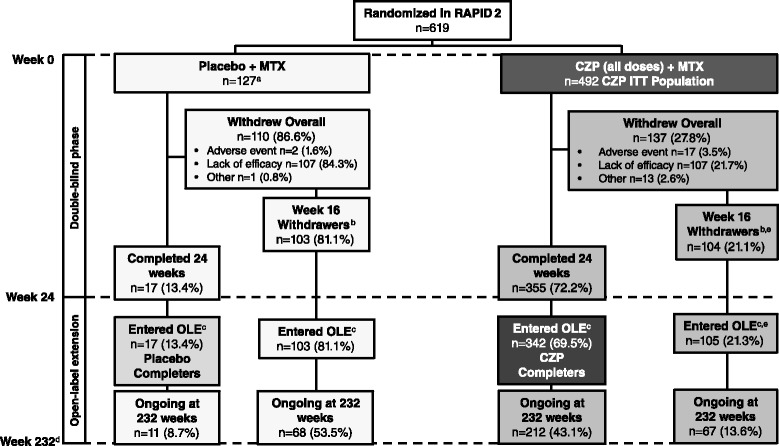
Patient disposition in the Rheumatoid arthritis prevention of structural damage (*RAPID*) 2 randomized controlled trial and open-label extension (*OLE*). Percentages were calculated by dividing number of patients by total number of patients in each treatment arm (n = 127 for placebo, n = 492 for certolizumab pegol (*CZP*)). ^a^Two patients randomized in the placebo group received CZP 200 mg in the double-blind phase, and were analyzed as part of the safety population; ^b^patients who withdrew due to lack of efficacy at week 16 were eligible to enter the OLE; ^c^at OLE entry all patients received 400 mg CZP + methotrexate every 2 weeks (Q2W), reduced to CZP 200 mg Q2W after ≥6 months; ^d^patients completed 232 weeks of CZP treatment from the first dose; ^e^number of withdrawers who entered the OLE differs from the number of week-16 withdrawers due to a windowing rule that resulted in a narrower group being defined for the week-16 withdrawers than actually were eligible for the OLE; week-16 withdrawers were defined as those who withdrew due to lack of efficacy at week 16, whereas withdrawers entering the RAPID 2 OLE included all withdrawers who entered the OLE, as recorded in the subject status evaluation. *AE* adverse event, *ITT* intention-to-treat, *MTX* methotrexate, *RCT* randomized controlled trial

In the RCT, 619 patients were randomized: 492 patients to 400 mg or 200 mg CZP + MTX (CZP ITT population) and 127 patients to placebo + MTX. In total, 355 CZP patients (72.2 %) and 17 placebo patients (13.4 %) completed the RCT, of which 342 CZP patients (week 24 CZP completer population) and all 17 placebo patients (week 24 placebo completer population) consented to enter the OLE at week 24. In addition to these, 207 patients entered the OLE at week 16 due to lack of ACR20 response, as per the study protocol. One further CZP patient withdrew after week 16 due to lack of ACR20 response and also entered the OLE, as a result of a windowing rule which caused a narrower group to be defined for the week 16 withdrawers than were actually eligible for the OLE (Fig. 1). Altogether, 567 patients from the RCT entered the OLE. Full patient disposition throughout the study is summarized in Fig. 1.

During the OLE, 369 patients in the CZP ITT underwent dose reduction to 200 mg Q2W (dose-reduction population). By week 232 of CZP treatment, 358 patients (57.8 % of all randomized patients) remained in the study. Overall, 612 patients received ≥1 dose of CZP in either the RAPID 2 RCT or OLE (safety population).

Baseline demographics and disease characteristics were comparable across all patient populations (CZP ITT, week 24 CZP completers, week 24 placebo completers, dose-reduction population and safety population; Table 1). However, disease duration in the week 24 placebo completers was approximately half that of the other patient populations (3.45 years).

**Table 1:** Baseline demographics and patient characteristics

Characteristic	CZP ITT population (n = 492)	Week 24 CZP completers (n = 342)	Week 24 placebo completers (n = 17)	Dose-reduction population (n = 369)	Safety population (n = 612)
Age, mean (SD), years	52.0 (11.4)	51.6 (11.6)	53.2 (16.0)	51.1 (11.3)	51.9 (11.5)
Gender, % female	80.9	82.7	82.4	81.8	81.5
Disease duration, mean (SD), years	6.3 (4.2)	6.5 (4.2)	3.5 (2.3)	6.2 (4.2)	6.2 (4.2)
RF-positive (≥14 IU/ml), %	76.5	75.4	88.2	73.7	76.9
Tender/painful joint count, mean (SD)	30.1 (14.2)	30.5 (14.5)	33.4 (12.1)	29.9 (13.9)	30.2 (14.0)
Swollen joint count, mean (SD)	20.7 (9.9)	20.7 (10.1)	24.2 (8.2)	20.6 (10.1)	21.0 (9.9)
Pain VAS, mean (SD)	61.2 (19.7)	60.6 (19.8)^a^	59.1 (21.6)	61.2 (19.5)^b^	60.9 (20.3)^c^
HAQ-DI, mean (SD)	1.6 (0.6)	1.6 (0.6)	1.6 (0.5)	1.6(0.6)	1.6 (0.6)^d^
DAS28(ESR), median (min, max)	6.8 (4, 9)^e^	6.8 (5, 9)^a^	7.1 (6, 8)	6.8 (5, 9)^b^	6.9 (4, 9)^f^

^a^n = 340; ^b^n = 367; ^c^n = 610; ^d^n = 611; ^e^n = 490; ^f^n = 608. *CZP* certolizumab pegol, *ITT* intention-to-treat; *RF* rheumatoid factor, *VAS* visual analog scale, *HAQ*-*DI* health assessment questionnaire-disability index, *DAS28* disease activity score 28, *ESR* erythrocyte sedimentation rate

### Patient retention

**Fig. 2:**
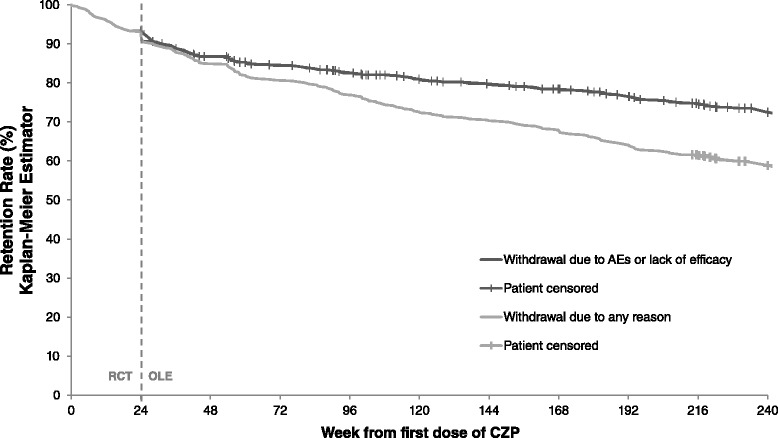
Kaplan-Meier plot of time to withdrawal for any reason and due to lack of efficacy or adverse event from start of the randomized controlled trial (*RCT*) for the certolizumab pegol (*CZP*) intention-to-treat (*ITT*) population (n =492). *AE* adverse event, *MTX* methotrexate, *OLE* open-label extension

Kaplan-Meier survival analyses estimated that 59.9 % of the CZP ITT population were retained to week 232, accounting for withdrawals for any reason (Fig. 2). If only withdrawals due to AEs or lack of efficacy were considered (i.e., patients withdrawing for other reasons were censored at time of discontinuation), the estimated retention rate after 232 weeks was 73.5 % (Fig. 2). The annual patient dropout rates throughout the study were 15.0 % for year 1, 11.2 % for year 2, 8.4 % for year 3 and 10.6 % for year 4 (Additional file 1: Table S1).

### Safety and exposure

Including the time in the RCT, the total median, mean and maximum durations of exposure to CZP were 1,326, 1,665 and 2,084 days (approximately 189, 238 and 298 weeks), respectively. AEs were reported in 546 out of 612 CZP-exposed patients (89.2 %) during the RCT and OLE (ER = 163.0) (Table 2). Most frequent AEs were upper respiratory tract infections, urinary tract infections and nasopharyngitis (Table 2). Active tuberculosis infection was reported in 6 patients (1.0 %) and herpes zoster infection in 10 patients (1.6 %). One pregnancy was reported during the study, which resulted in a spontaneous abortion.

**Table 2:** Summary of AEs and SAEs in the RAPID 2 RCT and OLE (safety population, n = 612)

	Patients, number (%)	Total number of events	Event rate per 100 PYs^a^	Incidence rate per 100 PYs
Total AEs	546 (89.2)	3789	163.0	87.4
Infections and infestations^b^	420 (68.6)	1257	54.1	36.4
Tuberculosis^c^	6 (1.0)	6	0.26	0.26
Herpes zoster^c^	10 (1.6)	11	0.47	0.43
Neoplasms (benign, malignant and unspecified)^b^	19 (3.1)	20	0.9	0.8
Most frequent AEs by preferred term (ER >4.0 per 100 PYs)				
Upper respiratory tract infections	93 (15.2)	154	6.6	4.4
Nasopharyngitis	67 (10.9)	101	4.3	3.1
Urinary tract infections	65 (10.6)	94	4.0	3.0
AEs leading to withdrawal	119 (19.4)	157	N/A	5.2
AEs leading to death by system organ class	19 (3.1)	25	N/A	0.8
Neoplasms (benign, malignant and unspecified)	5 (0.8)	5	N/A	0.2
Cardiac disorders	4 (0.7)	6	N/A	0.2
Nervous system disorders	4 (0.7)	4	N/A	0.2
Injury, poisoning and procedural complications	4 (0.7)	4	N/A	0.2
Infections and infestations	2 (0.3)	2	N/A	0.03
Hepatobiliary disorders	1 (0.2)	1	N/A	0.04
Musculoskeletal and connective tissue disorders	1 (0.2)	1	N/A	0.04
Total SAEs by system organ class	219 (35.8)	364	15.7	11.2
Infections/infestations	90 (14.7)	105	4.5	4.1
Musculoskeletal and connective tissue disorders	47 (7.7)	65	2.8	2.1
Neoplasms (benign, malignant and unspecified)	27 (4.4)	29	1.3	1.2
Injury, poisoning and procedural complications	23 (3.8)	25	1.1	1.0

^a^Event rates (ER) were not calculated when an adverse event (AE) occurred in individual patients only once (e.g., in the case of AEs leading to withdrawal or death); in these cases incidence rates are used; ^b^MedDRA system organ class; ^c^MedDRA preferred term. *n*/*a* not available, *PYs* patient-years, *SAE* serious adverse event

**Fig. 3:**
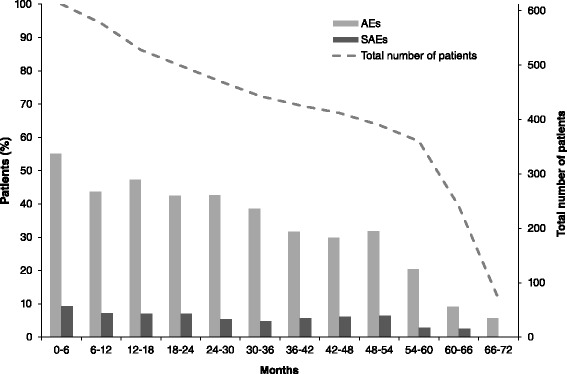
Percentage of patients experiencing adverse events (AEs) and serious AEs over each 6-month period from the start of the randomized controlled trial (safety population, n = 612). *SAE* serious adverse event

AE incidence was not constant over the whole treatment period (Fig. 3), which is the major assumption for the use of exposure-adjusted IRs [15, 16]. However, incidence of SAEs remained constant over the majority of the treatment period (Fig. 3). The overall ER for SAEs was 15.7 per 100 PYs (IR = 11.2). The most frequent SAEs (by MedDRA v9.0 system organ class) were serious infections and infestations (ER = 4.5, IR = 4.1), musculoskeletal and connective tissue disorders (ER = 2.8, IR = 2.1), benign, malignant and unspecified tumors (ER = 1.3, IR = 1.2) and injury, poisoning and procedural complications (ER = 1.1, IR = 1.0).

Overall, 119 patients (19.4 %) withdrew due to AEs (Table 2). There were 19 deaths (3.1 %) during the study, of which 13 were considered related to the study medication. As multiple AEs contributed to patient death, there were 25 fatal AEs in total. Fatal AEs by MedDRA system organ class (preferred term) included five malignancies (one event each of colon, gastric, pancreatic, gastrointestinal and lung malignancy; four considered related to the study drug), six cardiac disorders (two events of myocardial ischemia and one each of chronic cardiac failure, cardiopulmonary failure, ventricular arrhythmia and myocardial infarction; three related), four nervous system disorders (three events of cerebrovascular accidents and one cerebral hemorrhage; two related), two infections/infestations (one event each of toxic shock syndrome and tuberculosis of central nervous system; both related) one musculoskeletal and connective tissue disorder (one event of osteonecrosis; related), and one hepatobiliary disorder (one event of hepatic cirrhosis; related).

### Concomitant medication use

During the OLE portion of the study, the majority of patients who entered the OLE (n = 567) used concomitant NSAID medication (521 patients, 91.9 %), and just over two thirds of patients used corticosteroids (385 patients, 67.9 %) (Additional file 1: Table S2). DMARDs other than MTX were permitted in the OLE and were used by 133 patients (23.5 %) during the study. Very few patients used selective immunosuppressant agents or anti-malarial drugs (six patients, 1.1 % and two patients, 0.4 %, respectively).

### Clinical efficacy

**Fig. 4:**
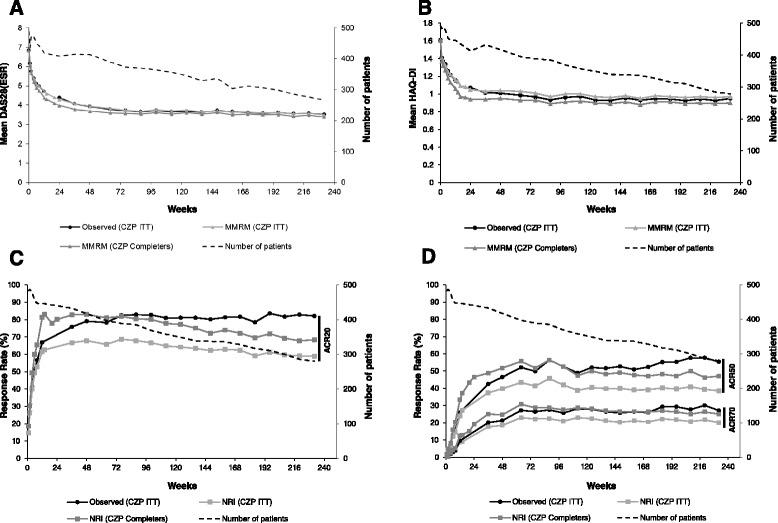
Efficacy variables in the certolizumab pegol (CZP) intention-to-treat (ITT) (n = 492) and week-24 CZP completer populations (n = 342). **a** Mean disease activity score in 28 joints (erythrocyte sedimentation rate) (DAS28(ESR)). **b** Mean health assessment questionnaire-disability index (HAQ-DI). **c** American College of Rheumatologists (ACR)20 response. **d** ACR50 and ACR70 responses. For patients who withdrew at week 16, observed data from the week-12 visit was also included in the week-24 data (start of open-label extension). *MMRM* mixed model repeated measures, *NRI* non-responder imputation

Improvements in disease activity (DAS28(ESR)) in the OLE were observed to week 232 in both the CZP ITT and week 24 CZP completer populations (Fig. 4a). Both populations had a mean DAS28(ESR) of 6.8 at RAPID 2 RCT baseline; after 36 weeks of CZP treatment this was reduced to 4.1 and 3.8, respectively, in the CZP ITT and CZP completers, and further reduced to 3.5 and 3.4 by week 232 (Fig. 4a, Additional file 1: Table S3A; MMRM imputation). After 232 weeks of CZP treatment, mean changes from baseline in DAS28(ESR) were −3.3 and −3.4, respectively (Additional file 1: Table S3B; MMRM imputation) and DAS28(ESR) remission rates were 14.1 % and 17.3 %. Observed DAS28(ESR) data and data imputed using MMRM were similar (Fig. 4a), as were data imputed using LOCF (Additional file 1: Figure S1).

ACR response rates observed during the RAPID 2 RCT were maintained until week 232 of the study (Fig. 4c and d). Although the true meaning of long-term ACR response over time is not fully clear, ACR20 was the primary endpoint and ACR50 and ACR70 response rates were secondary endpoints for the initial part of this study, therefore it is important to evaluate if these responses are stable over time. Week 232 ACR20, ACR50 and ACR70 response rates assessed by NRI were 58.9 %, 38.6 % and 20.1 % in the CZP ITT population and 68.4 %, 47.1 % and 25.1 % in the week-24 CZP completers. As expected, observed and MMRM response rates were higher than NRI-assessed ACR response rates (Additional file 1: Figure S1C and D). In the week-24 placebo completer population, NRI-assessed ACR20 response rates observed at week 36 (88.2 %) decreased by week 232 (64.7 %), whereas ACR50 and ACR70 response rates improved with long-term CZP treatment in the OLE from 47.1 % and 23.5 % at week 36 to 64.7 % and 35.3 % by week 232 (Additional file 1: Table S3C-E).

In the CZP ITT and week-24 CZP completer populations, similar percentages of patients achieved ACR-EULAR Boolean remission at week 232 (Boolean 4: CZP ITT = 7.5 %, CZP completers = 9.9 %; Boolean 3: CZP ITT = 8.5 %, CZP completers = 11.1 %) and SDAI and CDAI remission at week 232 (SDAI: CZP ITT = 10.2 %, CZP completers = 12.9 %; CDAI: CZP ITT = 10.4 %, CZP completers = 13.2 %).

#### Dose reduction

**Fig. 5:**
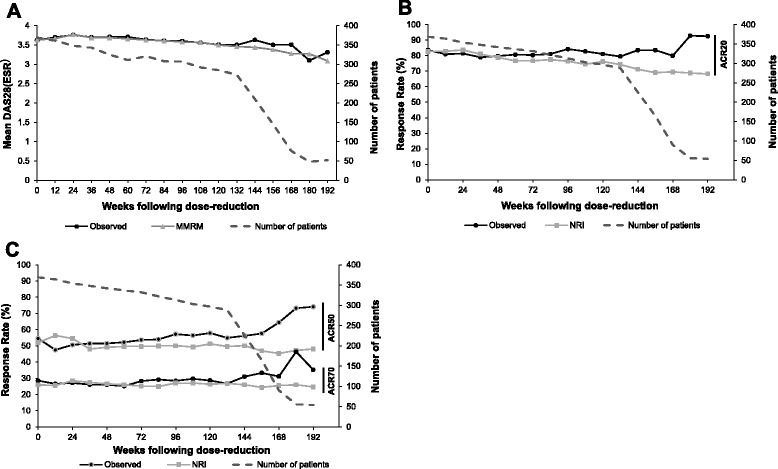
Efficacy variables in patients following dose reduction from certolizumab pegol (CZP) 400 mg every 2 weeks (Q2W) to CZP 200 mg Q2W (n = 369). **a** Mean disease activity score in 8 joints (erythrocyte sedimentation rate) (*DAS28*(*ESR*)). **b**American College of Rheumatology (*ACR*)20 response rates. **c** ACR50 and ACR70 response rates. This analysis is presented as weeks following dose-reduction visit (Week 0 is defined as the final efficacy assessment visit where a patient received the CZP 400 mg dose). The sharp decline in patient numbers from week 150 following dose reduction is due to the per-protocol site closures in countries where CZP became commercially available. *MMRM* mixed model repeated measures, *NRI* non-responder imputation

Clinical improvements were maintained following the reduction of CZP dose from 400 mg to 200 mg Q2W, the approved maintenance dose of CZP. At the dose-reduction visit, mean DAS28(ESR) was 3.6 in the dose-reduction population (n =369), which was sustained through 192 weeks of CZP 200 mg Q2W (Fig. 5a, MMRM imputation). Observed and LOCF DAS28(ESR) results were very similar to those imputed by MMRM (Additional file 1: Figure S2). ACR response rates were also maintained throughout the dose-reduction period (Fig. 5b and c).

#### Timing of response to CZP

When week-24 CZP completers were stratified by timing of response to CZP, there were 287 early week-12 ACR20 responders and 280 early DAS28 responders. At week 24, there were 30 late ACR20 responders and 47 late DAS28 responders. In total, 125 CZP-treated patients were ACR20 non-responders at OLE entry (Additional file 1: Table S4). Baseline characteristics were similar between early and late responder groups (Additional file 1: Table S4).

**Fig. 6:**
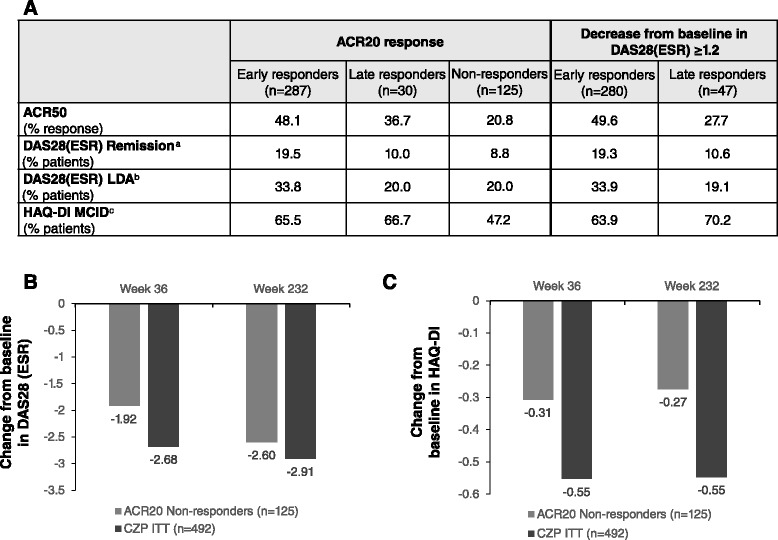
Comparison of clinical efficacy variables between patients responding early or late to certolizumab pegol (*CZP*), and American College of Rheumatology (*ACR*)20 non-responders who re-consented to the open-label extension study. **a** Percentage of patients achieving clinical outcomes at week 232 for early or late responders and ACR20 non-responders. **b** Change from baseline in mean disease activity score in 28 joints (erythrocyte sedimentation rate) (*DAS28*(*ESR*)) score in ACR20 non-responders compared to CZP intention-to-treat (*ITT*). **c** Change from baseline in mean health-assessment questionnaire-disability index (*HAQ*-*DI*) score in ACR20 non-responders compared to CZP ITT (last observation carried forward). Response was defined by ACR20 response or decrease from baseline in DAS28(ESR) ≥1.2. Early responders were those who responded to CZP at week 12 and late responders were those who responded to CZP at week 24 but not at week 12. ^a^Remission defined as DAS28(ESR) <2.6; ^b^low disease activity (*LDA*) defined as DAS28(ESR) ≤3.2; ^c^defined as a decrease from baseline of ≥0.22 in HAQ-DI. *MCID* minimal clinically important difference

In general, early responders had better long-term clinical outcomes than later responders (Fig. 6a). Higher ACR50 responses and better DAS28(ESR) scores were observed at week 232, with a greater percentage of early responders achieving DAS28(ESR) LDA or DAS28(ESR) remission (Fig. 6a). This was true when responses were defined by ACR20 response, or by decrease from baseline in DAS28(ESR) ≥1.2. In contrast, the percentage of patients reporting improvements in HAQ-DI ≥ the minimally clinically important difference (MCID) remained similar (Fig. 6a).

At week 232, 20.0 % of the late ACR20 responders achieved DAS28(ESR) LDA and 10.0 % achieved DAS28(ESR) remission compared with 20.0 % and 8.8 %, respectively, in the ACR20 non-responders (Fig. 6a). Changes from baseline in DAS28(ESR) scores at week 232 were also similar between the ACR20 non-responders and the CZP ITT population (−2.60 vs −2.91) (Fig. 6b).

When comparing the percentage of patients achieving ACR50 responses between ACR20 non-responders and ACR20 late responders, fewer ACR20 non-responders achieved this level of response at week 232 (20.8 % vs 36.7 %, respectively) (Fig. 6a). Similarly, fewer ACR20 non-responders reported clinically important HAQ-DI improvements at week 232 than late ACR20 responders (Fig. 6a). Lack of ACR20 responses at OLE entry also resulted in smaller improvements in HAQ-DI at week 232 compared to the entire CZP ITT population (Fig. 6c).

#### Radiographic progression

**Fig. 7:**
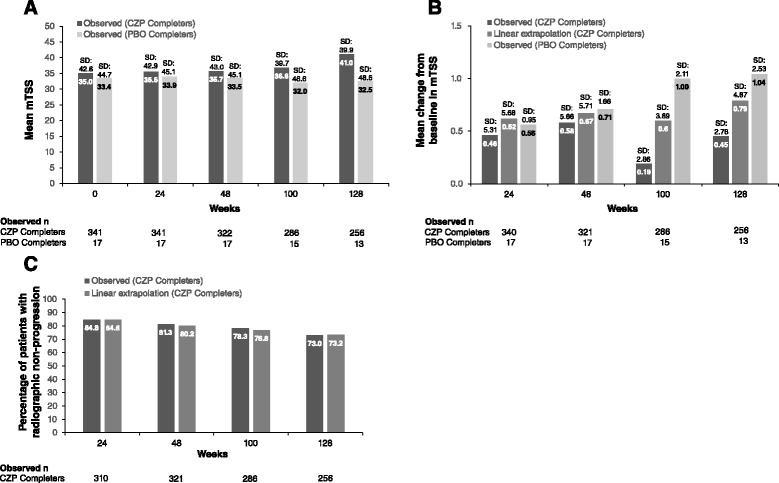
Assessment of structural damage in week-24 certolizumab pegol (*CZP*) and placebo (*PBO*) completers. **a** Mean modified total Sharp score (*mTSS*) (*Observed*). **b** Mean change from baseline in mTSS. **c** Percentage of patients with no radiographic progression. No radiographic progression was defined as an mTSS change from the randomized controlled trial baseline ≤0.5

At baseline, the observed mean mTSS score for the week-24 CZP completers was 33.4 (Fig. 7a); mean changes from baseline at week 24 and week 128 were 0.62 and 0.79, respectively (Fig. 7b, linear extrapolation). Even upon linear extrapolation of missing data, the mean progression over 2 years was approximately 0.5 mTSS units (Fig. 7b, linear extrapolation). The percentages of patients with no radiographic progression (defined as an mTSS change from RCT baseline ≤0.5) were 84.6 % at week 24 and 73.2 % at week 128 (Fig. 7c, linear extrapolation). In comparison, week-24 placebo completers had higher mTSS scores than week-24 CZP completers from baseline throughout the 2-year follow up period (Figs. 7a and b).

Analysis of the impact of timing of response on radiographic progression showed that long-term radiographic progression was similar whether patients responded to CZP at week 12 or week 24. In the early DAS28(ESR) responders (n = 280), the percentage of patients with no radiographic progression at week 128 was 72.7 %, compared with 76.9 % in the late DAS28(ESR) responders (n = 47).

### Physical function and health-related quality of life

**Fig. 8:**
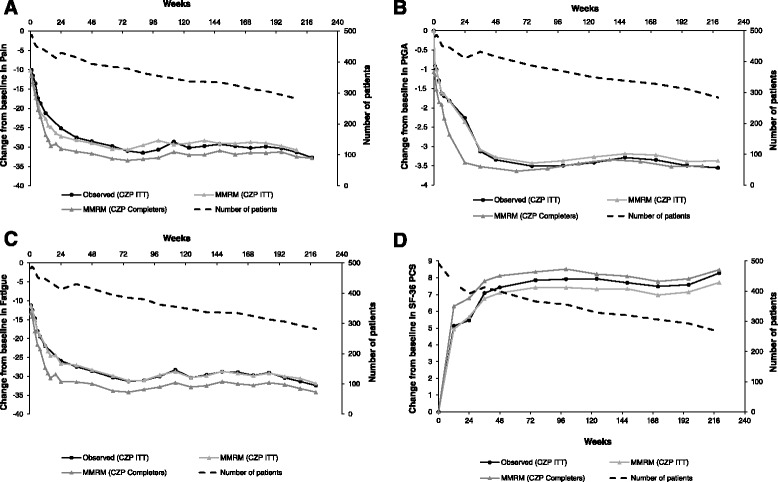
Patient-reported outcomes for certolizumab pegol (*CZP*) intention-to-treat (*ITT*) and week-24 CZP completer populations. **a** Change from baseline in patient’s assessment of arthritic pain. **b** Change from baseline in patient’s global assessment of disease activity (*PtGA*). **c** Change from baseline in fatigue. **d** Change from baseline in short form-36 physical component summary (*SF*-*36 PCS*). For patients who withdrew at week 16, observed data from the week-12 visit was also included in the week-24 data (start of open-label extension). *MMRM* mixed model repeated measures

**Fig. 9:**
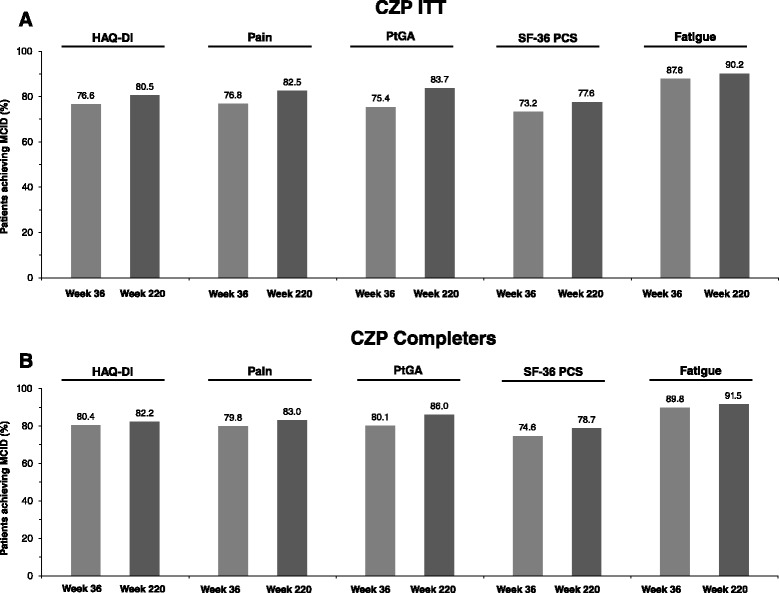
Improvements reported in minimally clinically important difference (*MCID*) for health-assessment questionnaire-disability index (*HAQ*-*DI*), patient’s assessment of arthritic pain, patient’s global assessment of disease activity (*PtGA*), short form-36 physical component summary (*SF*-*36 PCS*) and fatigue in certolizumab pegol (CZP) intention-to-treat (**a**) and CZP completers (**b**). Data were assessed using mixed model repeated measures imputation. MCID was defined as a decrease of ≥0.22 points from baseline in HAQ-DI, improvements ≥2.5 in the SF-36 PCS score from baseline, and for fatigue, pain and PtGA ≥10-point decrease from baseline. Data are presented at week 36 and week 220 as timing of visits meant that data were not available for week 24 and week 232

Improvements in PROs, including physical function, observed in the RAPID 2 RCT were maintained up to week 220 of CZP treatment (Fig. 4b, Fig. 8, and Additional file 1: Table S5). At 220 weeks, HAQ-DI scores decreased from a mean of 1.6 at RCT baseline to 0.96 and 0.89 in the CZP ITT and week-24 completer populations, respectively (Fig. 4b). Changes in pain, PtGA, fatigue and SF-36 PCS scores between RCT baseline and week 36 were also sustained during the OLE to week 220 (Fig. 8). Observed and MMRM-imputed data were similar, as were data imputed by last observation carried forward (LOCF) (Additional file 1: Figure S3). In both populations, the percentages of patients reporting improvements ≥MCID in HAQ-DI, pain, PtGA, SF-36 PCS and fatigue were maintained between weeks 36 and 220 (Fig. 9).

## Discussion

Treatment of RA patients with CZP + MTX over 5 years in this study revealed no new safety signals compared with the RAPID 1 evaluation of CZP + MTX, [17] and no increases in AEs or SAEs over time were observed. Patient retention was high in the RAPID 2 OLE, which indicates an acceptable tolerability profile and sustained efficacy of CZP. However, it should be noted that patient retention in clinical trials is often better than in real-world clinical practice. Clinical (ACR20/50/70, DAS28(ESR)) and patient-reported (HAQ-DI, PtGA, pain, fatigue, SF-36 PCS) improvements in RA signs, symptoms, and physical function observed over the 24-week RAPID 2 RCT were maintained for up to approximately 4.5 years in both the CZP ITT and week-24 CZP completer populations, consistent with sustained improvements observed in the RAPID 1 study [17]. Efficacy was also maintained in the dose-reduction population, demonstrating that CZP 200 mg Q2W + MTX was just as efficacious as CZP 400 mg Q2W + MTX during the maintenance phase.

Assessment of structural joint damage was documented for patients over 2 years of CZP treatment following the RAPID 2 RCT, and confirmed that TNF inhibition, here with CZP, can almost arrest long-term progression of joint damage in a majority of patients who completed the initial RCT. Radiographic progression in the week-24 CZP completers amounted to <1.0 point on the mTSS scale, which corresponds to an estimated irreversible disability increase of <0.01 in the HAQ-DI [18] over more than 2 years.

Although direct comparisons with other anti-TNFs are limited by differences in study design and lack of head-to-head studies, the observed incidences of SAEs and fatalities in the RAPID 2 OLE were comparable to long-term studies of other anti-TNFs [19–21]. Similarly, the IR of serious infections reported in this study (4.1/100 PYs) is consistent with IRs reported from OLE studies of other anti-TNFs (ranging from 2.8/100 PYs to 6.4/100 PYs) [19, 22–24] and also with a recent pooled safety analysis of clinical CZP trials in RA (3.65/100 PYs) [25]. In terms of efficacy, ACR response rates reported for the CZP completers at week 232 also appeared similar to long-term data reported for other anti-TNFs [19, 24, 26].

Numerically higher ACR50 response rates and lower DAS28(ESR) scores were observed in early responders compared to later responders, confirming the importance of an early response to CZP for some long-term clinical outcomes. These results are consistent with a previous post-hoc analysis of the RAPID 1 study, which demonstrated that faster attainment of clinical response to CZP was associated with improved long-term outcomes [27]. This is also in line with recent EULAR recommendations, which state that if no improvement is observed after 12 weeks of treatment, physicians should consider adjusting therapy [1]. However, in patients receiving CZP in the RCT, timing of response made little difference to the proportions of patients reporting improvements in HAQ-DI ≥MCID or experiencing radiographic progression. It should be noted that early response is likely important for long-term outcomes with other anti-TNFs and DMARDs, but this may not have been analyzed in as much detail as the RAPID 1 and 2 studies.

Lack of ACR20 response upon OLE entry appeared to be important for long-term ACR50 and HAQ-DI outcomes, but not for DAS28(ESR). A greater percentage of the later ACR20 responders achieved ACR50 responses and improvements in HAQ-DI ≥MCID compared with ACR20 non-responders, whereas the percentages of later ACR20 responders and ACR20 non-responders achieving DAS28(ESR) LDA and remission were similar. This is in line with previous notions on the limitations of the DAS28 [28, 29]. When compared to the CZP ITT population, ACR20 non-responders had similar long-term improvements in DAS28(ESR) scores, whereas lesser improvements were observed in HAQ-DI scores.

Limitations to this study included the relatively small number of patients who were evaluated. The drop-off in patient number towards the end of the study due to site closures in countries where CZP became commercially available made it impossible to report efficacy outcomes in patients with treatment duration ≥4.5 years. Additionally, exposure-adjusted IR was a pre-specified safety outcome in this study; however, the incidence of AEs was not constant over the treatment period, which is the main assumption for the use of exposure-adjusted IR [15, 16].

A major strength of this report relates to the method of assessment. Not only were data evaluated in patients who completed the RCT and consented to enter the OLE, but an ITT analysis was performed from the start of the trial, as requested by recent recommendations [30]. While, expectedly, the response rates were lower in the ITT than completer analyses, long-term maintenance of response to CZP was fully confirmed as an overall result.

## Conclusions

In the phase 3 multicenter RAPID 2 RCT and OLE, assessment of CZP as an additional medication to MTX in patients with active RA confirmed that long-term efficacy of CZP was sustained for almost 5 years, with no new safety signals observed compared to previous studies [17]. Efficacy was maintained following dose reduction from 400 mg to 200 mg Q2W, the approved dose of CZP, and early response to CZP at week 12 was again associated with improved long-term clinical outcomes. The long-term safety and efficacy of CZP reported here appears comparable to studies with other anti-TNFs [19–21, 24, 26], however, comparisons are limited due to differences in study design. These results not only confirm previously reported short-term data from the RAPID 2 RCT [2], they additionally support and expand the results from the RAPID 1 clinical trial and OLE [17].

## Additional files

Additional file 1: **Supplementary material.** Additional supplementary data tables and figures referred to in the manuscript. (PDF 1062 kb)

## Abbreviations

ACR: American College of Rheumatology
AEs: adverse events
CDAI: clinical disease activity index
CZP: certolizumab pegol
DAS: disease activity score
DMARD: disease-modifying anti-rheumatic drug
ER: event rate
ESR: erythrocyte sedimentation rate
EULAR: European League Against Rheumatism
HAQ-DI: health assessment questionnaire-disability index
IR: incidence rate
ITT: intention to treat
LDA: low disease activity
LOCF: last observation carried forward
LOE: lack of efficacy
MCID: minimally clinically important difference
MMRM: mixed model repeated measures
mTSS: modified total Sharp score
MTX: methotrexate
NRI: non-responder imputation
NSAID: non-steroidal anti-inflammatory drug
OLE: open-label extension
PCS: physical component summary
PRO: patient-reported outcome
PT: preferred term
PtGA: patient’s global assessment of disease activity
PYs: patient years
Q2W: every two weeks
RA: rheumatoid arthritis
RAPID: Rheumatoid arthritis prevention of structural damage
RCT: randomized controlled trial
SAEs: serious adverse events
SDAI: simplified disease activity index
SF-36: short-form 36 item health survey
SOC: system organ class
TEAEs: treatment-emergent adverse events
TNF: tumor necrosis factor
VAS: visual analog scale
