# Supplementary material for: Certolizumab pegol plus methotrexate 5-year results from the rheumatoid arthritis prevention of structural damage (RAPID) 2 randomized controlled trial and long-term extension in rheumatoid arthritis patients
Source: Arthritis Res Ther. 2015 Sep 10;17(1):245. doi: 10.1186/s13075-015-0767-2 (PMC4565002; doi:10.1186/s13075-015-0767-2)
Supplement: Supplementary file 1 — Supplementary material. Additional supplementary data tables and figures referred to in the manuscript. (PDF 1062 kb) [file 13075_2015_767_MOESM1_ESM.pdf]

SUPPLEMENTARY MATERIAL

Supplementary Table 1. Annual drop-out rate in the CZP ITT (n=492) during the RCT and OLE

Year of the study	Annual drop-out rate, %
Year 1	15.0
Year 2	11.2
Year 3	8.4
Year 4	10.6

CZP, certolizumab pegol; ITT, intent-to-treat; OLE, Open-Label extension; RCT, Randomized Controlled Trial.

Supplementary Table 2. Summary of concomitant medications taken by patients who entered the OLE (n=567)

Concomitant medication	n (%)
Any NSAID	521 (91.9)
Any corticosteroid	385 (67.9)
Any DMARD other than MTX	133 (23.5)
Any selective immunosuppressant agent	6 (1.1)
Any anti-malarial	2 (0.4)

DMARD, Disease Modifying Antirheumatic Drug; NSAID, Non-steroidal anti-inflammatory drug; OLE, Open-Label Extension.

Supplementary Table 3 Clinical efficacy outcomes at Week 36 and Week 232 for the CZP ITT population (n=492), Week 24 CZP Completers (n=342) and Week 24 Placebo Completers (n=17).

A. DAS28(ESR), mean (SD)

	CZP ITT population (N=492)		Wk 24 CZP Completers (n=342)		Wk 24 PBO Completers (n=17)	
Imputation method:	*LOCF*	*MMRM*	*LOCF*	*MMRM*	*LOCF*	*MMRM*
**Baseline**	6.83 (0.81)	6.82 (0.81)	6.83 (0.82)	6.82 (0.81)	7.02 (0.77)	7.02 (0.77)
**Week 36**	4.16 (1.31)	4.07 (1.21)	3.77 (1.16)	3.77 (1.14)	3.65 (1.20)	3.65 (1.20)
**Week 232**	3.92 (1.31)	3.50 (0.85)	3.70 (1.23)	3.39 (0.87)	3.55 (1.51)	3.13 (1.07)

B. DAS28(ESR), Change from baseline (SD)

	CZP ITT population (N=492)		Wk 24 CZP Completers (n=342)		Wk 24 PBO Completers (n=17)	
Imputation method:	*LOCF*	*MMRM*	*LOCF*	*MMRM*	*LOCF*	*MMRM*
**Week 36**	-2.68 (1.30)	-2.76 (1.21)	-3.07 (1.18)	-3.06 (1.16)	-3.38 (1.37)	-3.38 (1.37)
**Week 232**	-2.91 (1.37)	-3.07 (1.04)	-3.13 (1.36)	-3.37 (1.13)	-3.47 (1.70)	-3.89 (1.21)

C. ACR20, % response (95% CI)

	CZP ITT population (N=492)		Wk 24 CZP Completers (n=342)		Wk 24 PBO Completers (n=17)	
Imputation method:	*NRI*	*MMRM*	*NRI*	*MMRM*	*NRI*	*MMRM*
**Week 36**	66.7 (62.3, 70.8)	77.4 (73.5, 81.1)	82.7 (78.3, 86.6)	85.4 (81.2, 89.0)	88.2 (63.6, 98.5)	88.2 (63.6, 98.5)
**Week 232**	58.9 (54.5, 63.3)	85.2 (81.7, 88.2)	68.4 (63.2, 73.3)	88.0 (84.1, 91.3)	64.7 (38.3, 85.8)	82.4 (56.6, 96.2)

D. ACR50, % response (95% CI)

	CZP ITT population (N=492)		Wk 24 CZP Completers (n=342)		Wk 24 PBO Completers (n=17)	
Imputation method:	*NRI*	*MMRM*	*NRI*	*MMRM*	*NRI*	*MMRM*
**Week 36**	37.4 (33.1, 41.8)	39.8 (35.5; 44.3)	48.8 (43.4, 54.3)	50.0 (44.6, 55.4)	47.1 (23.0, 72.2)	47.1 (23.0, 72.2)
**Week 232**	38.6 (34.3, 43.1)	51.4 (46.9, 55.9)	47.1 (41.7, 52.5)	59.4 (53.9, 64.6)	64.7 (38.3, 85.8)	64.7 (38.3, 85.8)

E. ACR70, % response (95% CI)

	CZP ITT population (N=492)		Wk 24 CZP Completers (n=342)		Wk 24 PBO Completers (n=17)	
Imputation method:	*NRI*	*MMRM*	*NRI*	*MMRM*	*NRI*	*MMRM*
**Week 36**	17.7 (14.4, 21.3)	17.9 (14.6, 21.6)	25.1 (20.6, 30.1)	25.4 (20.9, 30.4)	23.5 (6.8, 49.9)	23.5 (6.8, 49.9)
**Week 232**	20.1 (16.7, 23.9)	20.9 (17.4, 24.8)	25.1 (14.2, 61.7)	26.6 (22.0, 31.6)	35.3 (20.6, 30.1)	23.5 (6.8, 49.9)

ACR, American College of Rheumatology; CI, Confidence Interval; CZP, certolizumab pegol; DAS (ESR), Disease Activity Score (Erythrocyte Sedimentation Rate); ITT, intent-to-treat; MMRM, Mixed Model Repeated Measures; PBO, Placebo; NRI, non-responder imputation; SD, Standard deviation; Wk, Week.

Supplementary Table 4 Baseline demographics and characteristics stratified by timing of response

Characteristic	ACR20 response			Decrease from baseline in DAS28(ESR) ≥1.2	
	Week 12 responders (n=287)	Week 24 responders (n=30)	Non-responders (n=125)	Week 12 responders (n=280)	Week 24 responders (n=47)
**Age, mean (SD), years**	51.5 (11.6)	52.9 (11.4)	52.1 (12.0)	51.2 (11.1)	54.6 (13.2)
**Gender, % female**	82.6	80.0	76.0	81.8	87.2
**Disease duration, mean (SD), years**	6.45 (4.22)	7.39 (3.67)	5.89 (4.18)	6.57 (4.17)	6.57 (4.62)
**Tender/painful joint count, mean (SD)**	30.4 (14.3)	33.0 (15.1)	30.5 (14.3)	31.7 (14.5)	25.4 (13.4)
**Swollen joint count, mean (SD)**	20.6 (9.9)	23.1 (12.2)	20.3 (9.9)	21.2 (10.3)	19.7 (10.0)
**Pain VAS, mean (SD)**	60.5 (20.0)	67.6 (16.6)	57.8 (19.0)	62.0 (19.4)	56.9 (21.3)
**HAQ-DI, mean (SD)**	1.6 (0.6)	1.7 (0.5)	1.6 (0.6)	1.6 (0.6)	1.6 (0.7)
**DAS28(ESR), median (min, max)**	6.8 (5, 9)	7.1 (5, 9)	6.6 (5, 9)	6.9 (5, 9)	6.4 (5, 8)

CZP, certolizumab pegol; DAS28, Disease activity score 28; HAQ-DI, Health assessment questionnaire-disability index; ITT, intent-to-treat; SD, standard deviation; VAS, visual analog scale.

Supplementary Table 5 Patient-reported outcomes at Week 36 and Week 220 for the CZP ITT population (n=492), Week 24 CZP Completers (n=342) and Week 24 Placebo Completers (n=17)

A. HAQ-DI, mean CFB (SD)

	CZP ITT population (N=492)		Wk 24 CZP Completers (n=342)		Wk 24 PBO completers (n=17)	
Imputation method:	*LOCF*	*MMRM*	*LOCF*	*MMRM*	*LOCF*	*MMRM*
**Week 36**	-0.55 (0.53)	-0.57 (0.51)	-0.65 (0.53)	-0.65 (0.53)	-0.78 (0.45)	-0.78 (0.45)
**Week 220**	-0.56 (0.63)	-0.62 (0.54)	-0.65 (0.63)	-0.69 (0.56)	-0.77 (0.63)	-0.81 (0.61)

B. Patient's Assessment of Arthritic Pain, mean CFB (SD)

	CZP ITT population (N=492)		Wk 24 CZP Completers (n=342)		Wk 24 PBO completers (n=17)	
Imputation method:	*LOCF*	*MMRM*	*LOCF*	*MMRM*	*LOCF*	*MMRM*
**Week 36**	-26.3 (24.0)	-27.2 (23.2)	-31.1 (24.0)	-31.1 (23.5)	-33.5 (22.4)	-33.5 (22.4)
**Week 220**	-26.9 (26.7)	-30.7 (23.0)	-30.2 (27.4)	-32.9 (23.8)	-29.8 (21.8)	-30.5 (20.1)

C. PtGA, mean CFB (SD)

	CZP ITT population (N=492)		Wk 24 CZP Completers (n=342)		Wk 24 PBO completers (n=17)	
Imputation method:	*LOCF*	*MMRM*	*LOCF*	*MMRM*	*LOCF*	*MMRM*
**Week 36**	-26.3 (24.1)	-27.0 (23.5)	-31.5 (23.6)	-31.5 (23.2)	-34.7 (23.1)	-34.7 (23.1)
**Week 220**	-26.8 (27.1)	-31.9 (22.4)	-30.5 (27.5)	-34.2 (22.5)	-30.5 (28.1)	-31.9 (26.4)

D. Fatigue, mean CFB (SD)

	CZP ITT population (N=492)		Wk 24 CZP Completers (n=342)		Wk 24 PBO completers (n=17)	
Imputation method:	*LOCF*	*MMRM*	*LOCF*	*MMRM*	*LOCF*	*MMRM*
**Week 36**	-2.9 (2.3)	-3.1 (2.2)	-3.4 (2.2)	-3.4 (2.2)	-3.5 (2.5)	-3.5 (2.5)
**Week 220**	-2.9 (2.4)	-3.4 (2.1)	-3.2 (2.5)	-3.5 (2.2)	-3.5 (2.6)	-3.8 (2.1)

E. SF-36, mean CFB (SD)

	CZP ITT population (N=492)		Wk 24 CZP Completers (n=342)		Wk 24 PBO completers (n=17)	
Imputation method:	*LOCF*	*MMRM*	*LOCF*	*MMRM*	*LOCF*	*MMRM*
**Week 36**	6.7 (7.8)	6.8 (7.5)	7.9 (8.2)	7.8 (8.0)	9.8 (7.3)	9.82 (7.3)
**Week 220**	6.9 (8.8)	7.7 (7.6)	7.7 (9.1)	8.5 (8.1)	9.7 (9.4)	10.9 (9.3)

CFB, change from baseline; CZP, certolizumab pegol; HAQ-DI, Health-Assessment Questionnaire-Disability Index; ITT, intent-to-treat; LOCF, Last Observation Carried Forward; MMRM, Mixed Model Repeated Measures; PBO, Placebo; PtGA, Patient's global assessment of disease activity; SD, Standard Deviation;SF-36 PCS, Short Form-36 Physical Component Summary; Wk, Week.

Supplementary Figure 1 Efficacy variables for CZP ITT population and Week 24 CZP Completers. (A) Mean DAS28 (ESR) (Observed, LOCF and MMRM) (B) Mean HAQ-DI (Observed, LOCF and MMRM) (C) ACR20 response (Observed, NRI and MMRM) (D) ACR50 and ACR70 responses (Observed, NRI and MMRM)

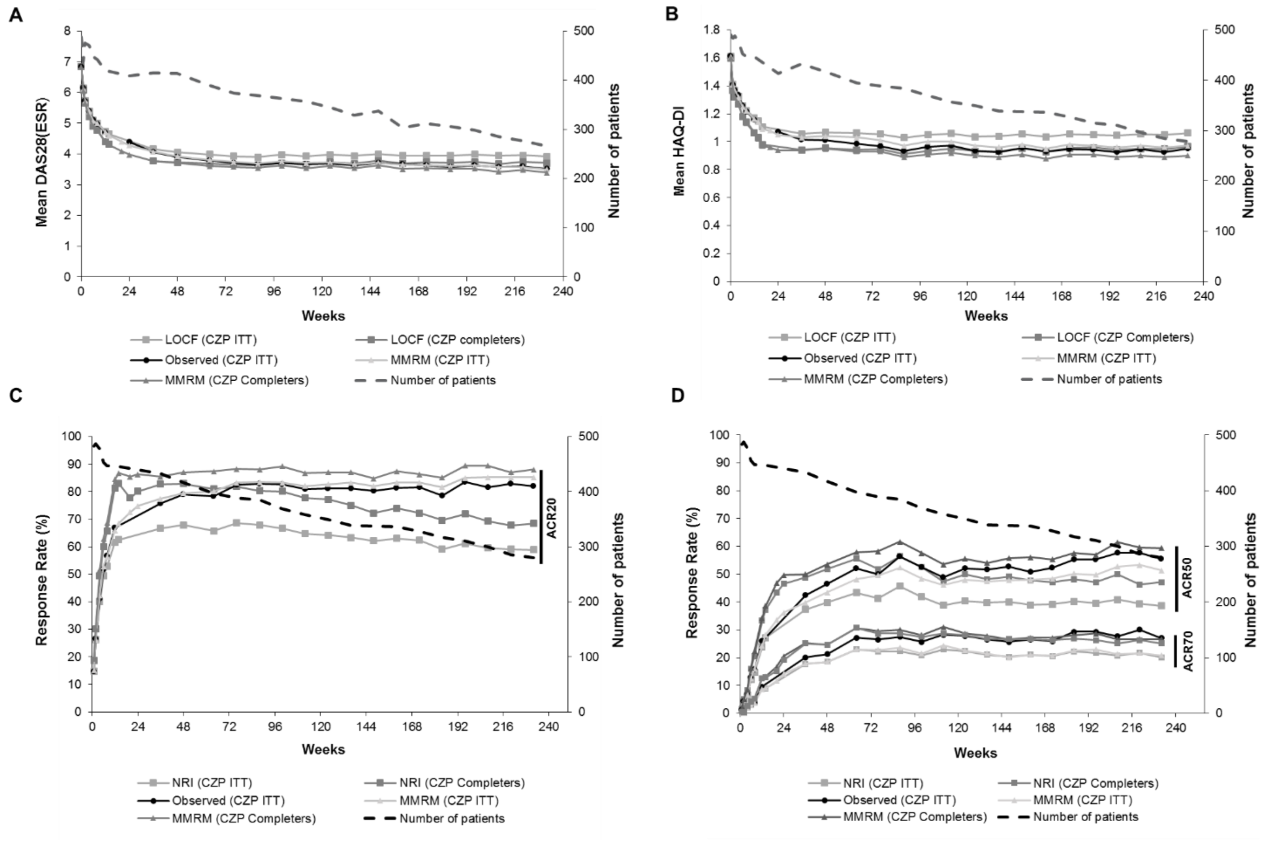

ACR, American College of Rheumatology; CI, Confidence Interval; CZP, certolizumab pegol; DAS (ESR), Disease Activity Score (Erythrocyte Sedimentation Rate); ITT, intent-to-treat; LOCF, Last Observation Carried Forward; MMRM, Mixed Model Repeated Measures; NRI, non-responder imputation.

Supplementary Figure 2 Efficacy variables in patients following dose-reduction from CZP 400 mg Q2W to CZP 200 mg Q2W. (A) Mean DAS28(ESR) (Observed, LOCF and MMRM), (B) ACR20 response rates (Observed, NRI and MMRM) (C) ACR50 and ACR70 response rates (Observed, NRI and MMRM)

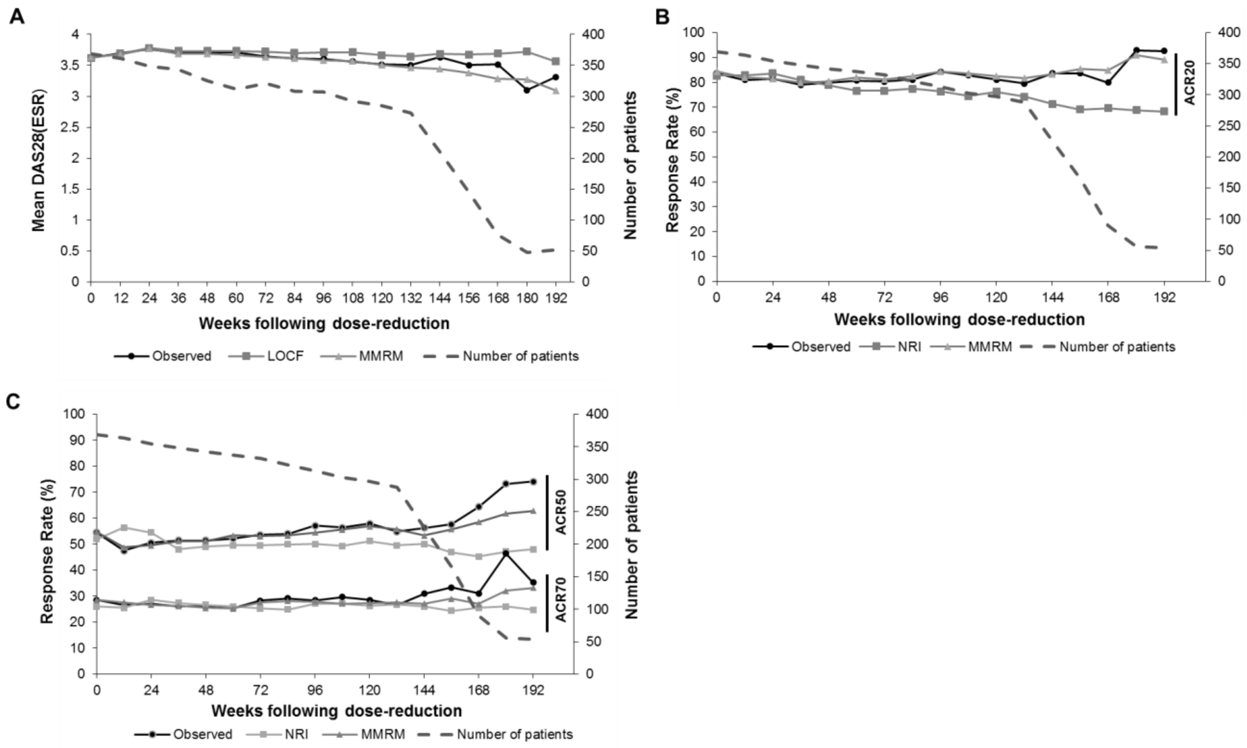

For patients who withdrew at Week 16, observed data from the Week 12 visit was also included in the Week 24 data (start of open-label extension). ACR, American College of Rheumatology; CZP, certolizumab pegol; DAS28(ESR), Disease Activity Score (Erythrocyte Sedimentation Rate), LOCF, Last Observation Carried Forward; MMRM, Mixed Model Repeated Measures, NRI, Non-responder Imputation; Q2W, every other week.

Supplementary Figure 3 Patient-reported outcomes for CZP ITT and CZP Completer populations (Observed, LOCF and MMRM) (A) Change from baseline in Patient's assessment of arthritic pain (B) Change from baseline in PtGA (C) Change from baseline in Fatigue (D) Change from baseline in SF-36 PCS

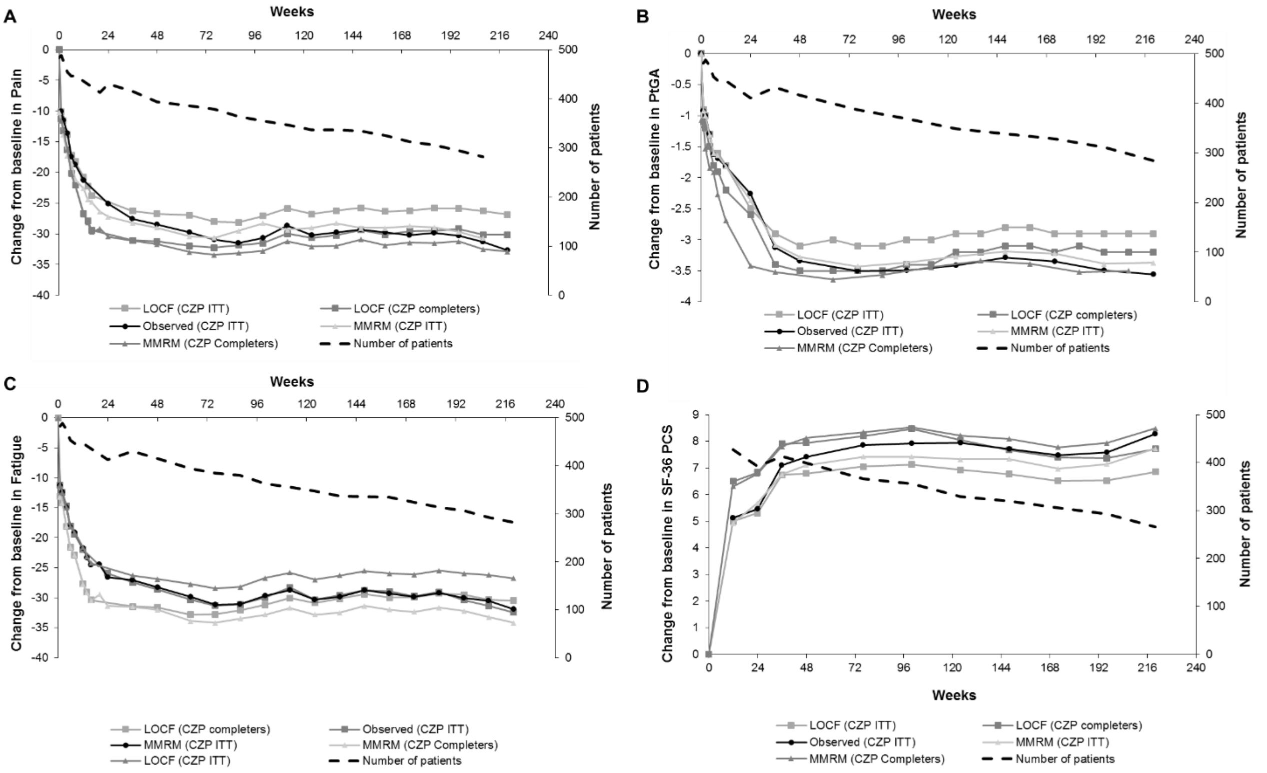

For patients who withdrew at Week 16, observed data from the Week 12 visit was also included in the Week 24 data (start of open-label extension). CZP, certolizumab pegol; HAQ-DI, Health Assessment Questionnaire-Disability Index; ITT, intent-to-treat; LOCF, Last Outcome Carried Forward; MMRM, Mixed Model Repeated Measures; PtGA, Patient's Global Assessment of disease activity; SF-36 PCS, Short Form-36 Physical Component Summary.

Supplementary Figure 4 Percentage of patients reporting improvements in MCID (LOCF/NRI and MMRM) for HAQ-DI, Patient's Assessment of Arthritic Pain, PtGA, SF-36 PCS and Fatigue in (A) CZP ITT and (B) Week 24 CZP Completers

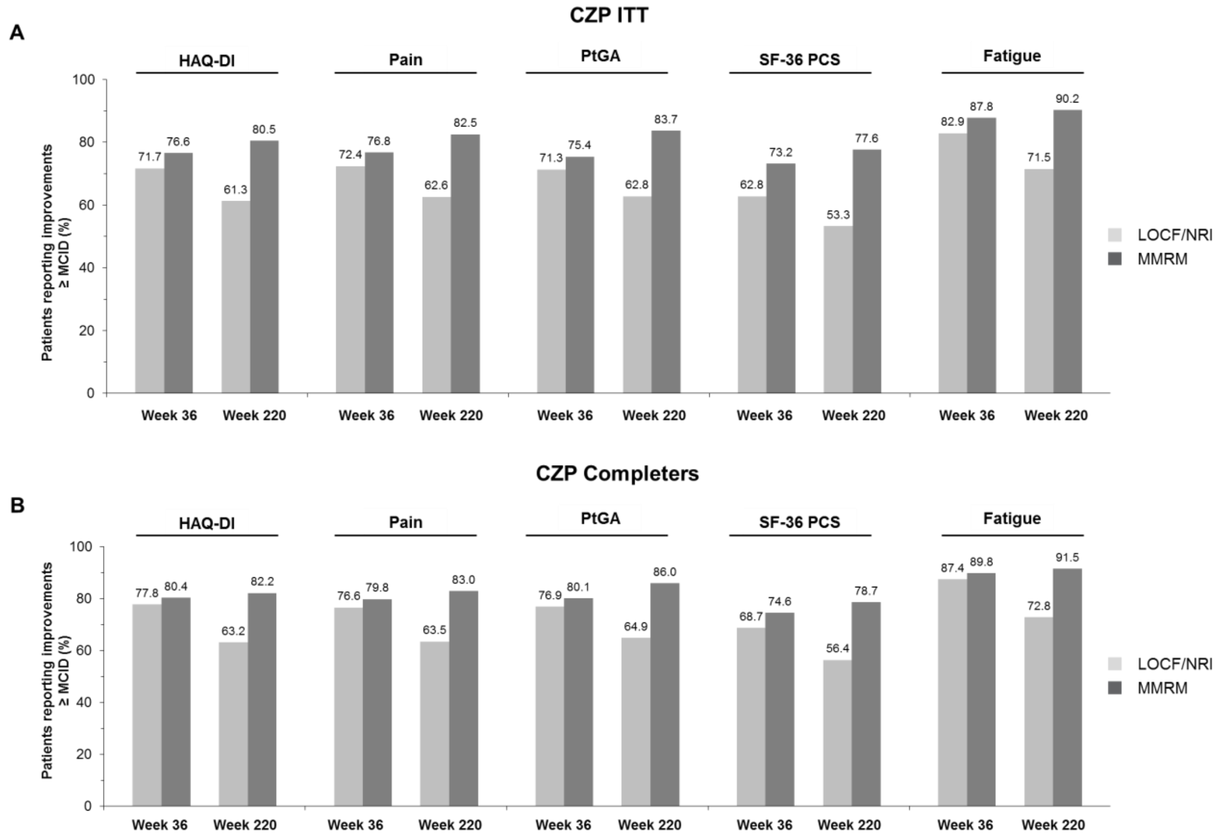

Minimal clinically important differences (MCID) were defined as a decrease of ≥0.22 points from baseline in HAQ-DI, improvements ≥2.5 in the SF-36 physical component summary (PCS) score from baseline, and for Fatigue, Pain and PtGA ≥10 point decrease from baseline. Data are presented at Week 36 and Week 220 as timing of visits meant that data was not available for Week 24 and Week 232. For LOCF/NRI imputation, missing values during the RCT were imputed by carrying forward the last available post-baseline composite score. For patients entering the OLE, missing values during the OLE because of patient withdrawal due to adverse event or lack of efficacy or because of data exclusion after the use of rescue medication before that visit were imputed as not reaching the MCID criterion whereas missing values because of other reasons for withdrawal, study completion or because of a missing assessment were imputed by carrying forward the last available post-entry score. If still missing (eg. no suitable value available for LOCF) then value was imputed as not reaching MCID criterion. For patients not entering the extension study, missing values were imputed by carrying forward the last available post-baseline composite score. HAQ-DI, Health-Assessment Questionnaire-Disability Index; ITT, intent-to-treat; MMRM, Mixed Model Repeated Measures; NRI, Non-responder Imputation; PtGA, Patient's Global Assessment of disease activity; SF-36 PCS, Short Form-36 Physical Component Summary
